# Supplementary material for: Identification of Terpenoid Compounds and Toxicity Assays of Essential Oil Microcapsules from Artemisia stechmanniana
Source: Insects. 2023 May 16;14(5):470. doi: 10.3390/insects14050470 (PMC10231053; doi:10.3390/insects14050470)
Supplement: Supplementary file 1 [file insects-14-00470-s001.zip › insects-2377081-supplementary.pdf]

Table S1. Box-Behnken Design factors and level for microencapsule

Plants essential oil	Core/Shell mass ratios	Embedding rate (%)	Loading rate (%)
*Artemisia mongolica**	1:8	68	8.5
*Artemisia mongolica**	1:6	48	6.8
*Artemisia mongolica**	1:10	55	7.4
*Artemisia stechmanniana***	1:8	52	8.6

* Modified from Liu C., Liu Z.L., Zhang R., Huang W.G., Wang F., Quan M.R. (2022) Control Effect of the Essential Oil of *Artemisia mongolica* against Three Main Pests of Wolfberry (*Lycium barbarum*). Chinese Journal of Biological Control, 1-11.

** microencapsule in this study.

Table S2. GC–MS analys relative proportion (in %) of main components from *A. stechmanniana* and *A. mongolica* EOs in the terpenoid compounds

	Eucalyptol	(+)-2-Bornanone	1-(1,2,3-Trimethyl-cyclopent-2-enyl)-ethanone	(-)-Spathulenol	Others
A. stechmanniana	15.84	16.92	25.63	16.38	25.26
A. mongolica	28.24	3.49	0	0.45	67.82

Table S3. Cotronl efficacy (CE%) of *A. stechmanniana* essential oil to three different insect species in the *Lycium barbarum* field

Insects	Days	Compound	Control efficacy	CE
A. gossypii	1	Dose I	53.54±1.30	M
	3		54.26±2.61	M
	7		60.54±1.17	S
	14		50.66±0.90	M
	21		40.39±1.44	M
F. occidentalis	1	Dose I	51.98±2.02	M
	3		53.73±1.55	M
	7		33.3±1.38	M
	14		41.65±0.93	M
	21		41.65±0.93	M
B. gobica	1	Dose I	59.13±1.37	M
	3		54.65±1.81	M
	7		67.16±1.43	S
	14		45.22±0.80	M
	21		43.92±3.63	M
A. gossypii	1	Dose II	71.27±0.84	S
	3		71.21±0.56	S
	7		66.30±1.69	S
	14		52.51±2.06	M
	21		55.60±1.74	M
F. occidentalis	1	Dose II	53.98±0.88	M
	3		56.48±2.37	M
	7		38.35±0.97	M
	14		44.54±2.12	M
	21		44.54±2.12	M
B. gobica	1	Dose II	60.88±2.21	S
	3		70.11±0.70	S
	7		71.19±0.79	S
	14		54.20±0.98	M
	21		46.92±2.08	M
A. gossypii	1	Dose III	76.91±1.24	S
	3		75.87±1.90	S
	7		73.16±0.67	S
	14		51.83±1.35	M
	21		52.67±1.34	M
F. occidentalis	1	Dose III	60.64±0.86	S
	3		62.39±1.20	S
	7		45.31±2.03	M
	14		51.17±0.93	M
	21		51.17±0.93	M
	1	Dose III	72.00±1.95	S
	3		76.56±0.59	S

B. gobica	7	83.60±2.00	S
	14	62.38±1.84	S
	21	55.14±1.27	M

CE, Control efficacy. S, strong (CE > 60%); M, moderate (20% < CE <60%); Dose I: 129 g a.i./hm2; Dose II: 258 g a.i./hm2; Dose III: 516 g a.i./hm2.
